# Systemic Lupus Erythematosus Presenting as Anorexia Nervosa in a Middle‐Aged Woman: A Rare Case Report and Literature Review

**DOI:** 10.1002/iid3.70083

**Published:** 2025-01-27

**Authors:** Maen Nizam Baroudi, Anwar I. Joudeh, Mohammed Kays Alattiya, Abdo Qaid Ahmed Lutf, Hassan Abuzaid, Salah Mahdi

**Affiliations:** ^1^ Department of Internal Medicine Al‐Khor Hospital, Hamad Medical Corporation Doha Qatar; ^2^ Department of Internal Medicine Collage of Medicine Qatar University Doha Qatar

**Keywords:** anorexia nervosa, case report, Graves's disease, neuropsychiatric lupus, systemic lupus erythematosus

## Abstract

**Background:**

Systemic lupus erythematosus (SLE) is a complex autoimmune disease with multisystemic involvement and unclear etiology. Although SLE could be linked to multiple neuropsychiatric manifestations, the co‐occurrence of anorexia nervosa was only described through a few case reports that mainly affected children and adolescents.

**Case Presentation:**

a 40‐year‐old Filipina woman presented to hospital with a 3‐day history of agitation, anorexia and auditory hallucinations. She also had restrictive dietary habits with substantial weight loss and excessive fear of weight gain for the past several months. Her medical history was also noticeable for a 3‐month history of erythematous non‐itchy skin rash. On examination, the patient was pale and underweight. She had angular stomatitis, bilateral exophthalmos, and diffuse goiter. Skin examination revealed diffuse scaly and erythematous rash over the neck and upper torso with post‐inflammatory skin discoloration. Laboratory investigations showed pancytopenia, high serum creatinine, and +1 proteinuria. Further workup confirmed thyrotoxicosis due to Graves disease as well as having multiple positive autoantibodies including antinuclear antibody (ANA) which were suggestive for SLE. A subsequent kidney biopsy demonstrated class IV diffuse lupus nephritis. The patient was treated successfully with intravenous pulse steroid therapy, rituximab, and antithyroid medications with no residual symptoms or laboratory abnormalities.

**Conclusions:**

The concurrence of anorexia nervosa with SLE and the complete resolution of anorexia nervosa symptoms with immunosuppressive therapy suggest common autoinflammatory pathogenesis for both conditions. Further research is needed to elucidate any potential association between SLE and anorexia nervosa.

**Abbreviations**
ACR: American College of Rheumatology
CSF: cerebrospinal fluid
DSM‐5‐TR: Diagnostic and Statistical Manual of Mental Disorders, Fifth Edition, Text Revision
MRI: magnetic resonance imaging
NPSLE: neuropsychiatric systemic lupus erythematosus
SLE: systemic lupus erythematosus
SLICC: Systemic Lupus International Collaborating Clinics diagnostic criteria

## 1 Background

Systemic lupus erythematosus (SLE) is a multisystemic disease with a wide range of clinical manifestations and unknown etiology. It could potentially affect any organ in the body with a variable severity ranging from simple skin or joint involvement to life‐threatening renal failure, bone marrow suppression, or neurological impairment. SLE is a heterogenous syndrome that likely results from complex multifactorial interactions between genetics, environmental, and hormonal factors. Moreover, the absence of pathognomonic clinical findings or laboratory investigations makes the diagnosis of SLE more challenging for the treating physician [1]. The association of SLE with other autoimmune thyroid diseases particularly Hashimoto thyroiditis has been frequently described with fewer reports on Graves' disease [2, 3]. However, the co‐occurrence of SLE with anorexia nervosa was only described through a few case reports and small case series affecting mainly the pediatric population [4, 5, 6, 7, 8].

Anorexia nervosa is a unique form of restrictive eating disorder where patients who are abnormally underweight suffer from abnormal perception of their body weight and shape, and excessive fear of weight gain [9]. Although sociocultural factors may play a role in the onset of this eating disorder, it is increasingly recognized that immunopathological mechanisms and genetic susceptibility predispose individuals to the development of anorexia nervosa [10]. Furthermore, the bidirectional relationship between anorexia nervosa and autoimmune disorders could be explained by shared immunological pathways that result in abnormal T‐cell tolerance and autoantibody formation [10, 11]. Herin, we describe a case of a 40‐year‐old woman who presented to our hospital with symptoms of anorexia nervosa and Graves' disease who later developed a cascade of SLE criteria. Despite the myriad of SLE complications that the patient had, she recovered completely with no residual psychiatric or renal manifestations.

## 2 Case Presentation

A 40‐year‐old Filipina care worker was brought to the emergency department by a friend due to aggressive behavior, agitation, and refusal to eat for the past 3 days. She also reported experiencing auditory hallucinations and recurrent episodes of feeling of hotness, accompanied by shivering and palpitations. Over the past several months, her friend observed significant weight loss, along with increasing fatigue, pallor, irritability, and social withdrawal, particularly from events involving food. The patient frequently skipped meals and, despite reassurances from her friend that her weight was normal, remained convinced she was overweight and was deeply preoccupied with her body image. She obsessively counted calories and, even after eating small portions, would take antiemetics and over‐the‐counter laxatives to prevent weight gain. Over the past year, she lost approximately 15 kg, experienced oligomenorrhea, and suffered from excessive hair loss and weakened nails.

She also had a 3‐month history of skin rash for which she used over‐the‐counter topical treatment. The rash was mainly involving the upper part of her body and it was not itchy. However, healing of the lesions was followed by skin darkening.

The patient denied any history of headache, blurring of vision, or abnormal movement. There were no recent stressful events and no previous history of any psychiatric disorder or illicit drug abuse. No previous history of chronic medical illness and she was not using any regular medications. The patient denied smoking or alcohol intake, and her family history was negative for psychiatric illness or autoimmune diseases.

On examination, the patient was agitated with increased motor activity, had poverty of speech, perplexed affect, and delusion of persecution. She also was pale and underweight with a body mass index (BMI) of 17 Kg/m^2^. Her pulse rate was 145 beats per minute, and her respiratory rate was 24/minute. Head and neck examination revealed diffuse nonscarring alopecia, bilateral exophthalmos, angular stomatitis, and diffuse goiter. She also had bilateral fine hand tremors and multiple areas of erythematous scaly and hyperpigmented rash in the neck, both arms and upper torso. There was tenderness over both wrists and knees without swelling or redness. However, the rest of the physical examination was unremarkable.

Laboratory investigations showed severe anemia (hemoglobin 4.6 gm/dL [12.5–16 gm/dL]), thrombocytopenia (platelets count 92 × 10^3^/μL [150–410 × 10^3^/μL]), high serum creatinine (196 μmol/L [44–80 μmol/L]) and high urea (22.4 mmol/L [2.5–7.5 mmol/L]) as well as low total serum protein (53 gm/L [60–80 gm/L]) and albumin (13 gm/L [35–50 gm/L]). The urine dipstick was positive for RBCs, WBCs and +1 protein, and the urine protein‐creatinine ratio was 144 mg/mmol.

The thyroid function test was consistent with primary hyperthyroidism with a suppressed TSH ( < 0.01 mIU [0.3–4.2]) and high free T4 (33.8 pmol/L [11–23 pmol/L]). The anti‐TSH receptor antibody, anti‐thyroid peroxidase antibody and anti‐thyroglobulin antibody titers were all positive in high titers ( > 40 IU/L, > 600 IU/mL, and 1205 IU/mL respectively). In addition, the patient had high antinuclear antibody (ANA) titer (1:1320 speckled pattern), with positive anti ds‐DNA antibody (> 379 IU/mL), anti‐Sm antibody (> 480 U/mL), anti‐RNP antibody (> 240 U/mL), anti‐Scl‐70 antibody (46 U/mL) with a low C3 and C4 levels (0.23 and 0.2 gm/L respectively). The N‐terminal pro‐b‐type natriuretic peptide (NT pro‐BNP) level was very high at 40,407 pg/mL.

The patient underwent brain magnetic resonance imaging (MRI) and a lumbar puncture and they were both negative. The cerebral fluid (CSF) analysis included testing for CSF cell blood count, total protein, glucose, gram stain and culture, cryptococcal antigen, acid‐fast bacilli smear, mycobacterial tuberculosis PCR, culture, and oligoclonal bands. An ultrasound examination of the thyroid showed a diffusely enlarged gland with hypervascularity. An ultrasound‐guided kidney biopsy showed diffuse lupus nephritis, ISN/RPS class IV, and echocardiography showed mildly reduced left ventricle ejection fraction (44%) with grade II diastolic dysfunction and severe pulmonary hypertension (resting right ventricular systolic pressure of 62 mmHg).

Based on the above, the patient was diagnosed with SLE with multiorgan involvement, anorexia nervosa and Grave's disease. She was managed under the care of a multidisciplinary team of a rheumatologist, nephrologist, cardiologist, endocrinologist, psychiatrist, psychotherapist, specialized nurses, and dietitian. She was treated initially with methylprednisolone 250 mg intravenously once daily for 3 days followed by a tapering dose of oral prednisolone (starting from 30 mg once daily) with hydroxychloroquine 200 mg once daily and mycophenolate mofetil (500 mg twice daily). She received a 1 g dose of rituximab infusion, followed by a second dose 2 weeks later, and then 1 g every 6 months. In addition, she was started on carbimazole (15 mg twice daily) and propranolol (40 mg twice daily) with eventual restoration of euthyroid status.

Hospitalization course was complicated by a brief period of fluid overload due to worsening renal parameters that required few sessions of hemodialysis. However, the patient continued to improve clinically and biochemically and her kidney function improved after 1 month and we stopped the hemodialysis. At a follow‐up visit 9 months later, the patient was feeling better. She denied having a recurrence of auditory hallucination and her appetite improved. There was no joint or skin rash and the alopecia improved markedly. Her BMI improved to 23 Kg/m^2^. The laboratory investigations showed normal full blood counts and serum urea and creatinine levels. The urine protein creatinine ratio improved to 30 mg/mmol. The anti‐ds‐DNA antibody titer became negative and the C3 and C4 levels went back to the normal range. The patient continued to follow up regularly in our rheumatology clinic and remained in clinical remission. Up to this date (19 months post‐diagnosis), the patient did not have recurrence of anorexia nervosa symptoms and her BMI increased to 25 Kg/m2. Her latest laboratory investigations showed normal full blood count, renal and liver function tests with negative proteinuria. she received only one dose of maintenance rituximab around 1 year ago, and her current medications are hydroxychloroquine 200 mg twice daily, azathioprine 100 mg once daily, valsartan 80 mg once daily, metoprolol 50 mg twice daily and carbimazole 20 mg twice daily.

## 3 Discussion and Conclusions

SLE was diagnosed based on seven clinical criteria from the 2012 Systemic Lupus International Collaborating Clinics (SLICC) diagnostic criteria: subacute cutaneous lupus, nonscarring alopecia, neuropsychiatric symptoms, renal involvement, anemia, leukopenia, and thrombocytopenia. Immunologically, the patient had positive ANA, anti‐dsDNA, Anti‐Sm, and hypocomplementemia [12]. Anorexia nervosa was diagnosed using DSM‐5‐TR criteria and classified as mild, with a BMI of 17 kg/m² [9].

SLE, a highly variable disease, frequently includes neuropsychiatric manifestations with a prevalence of 20%–40%, which can occur at any stage of the disease. These events, though possibly unrelated to lupus pathology, are linked to higher mortality and poorer quality of life [13, 14]. The American College of Rheumatology (ACR) has identified 19 neuropsychiatric syndromes in SLE, but anorexia nervosa is not one of them [15].

We conducted a literature review in the PubMed database for original reports describing the coincidence of anorexia nervosa in patients with SLE using the keywords: SLE AND Anorexia nervosa NOT review with no specified date. We identified 4 case reports [4, 6, 7, 8], and one case series for seven children and adolescents [5]. One case report was excluded because we could not retrieve the full text [6]. The extracted case reports and series are summarized in Table 1 (total number 10 patients). The majority were young females aged 13–16 years, with anorexia nervosa diagnosed before or near SLE diagnosis. In two cases, anorexia nervosa developed after SLE, attributed to steroid use [5, 8]. All patients were ANA‐positive, and many had hematological involvement. Fever, malar rash, arthritis, and Raynaud's phenomenon were common [4, 7, 16]. A later case report that was published after completing the literature review described a 22‐year‐old man with food restriction before SLE diagnosis, who improved with psychiatric care and SLE treatment [16].

**Table 1:** Summary of the case reports and case series on concomitant anorexia nervosa with systemic lupus erythematosus.

Authors [Reference]	Age	Sex	Onset of anorexia nervosa	Affected organs with SLE	Treatment and outcome
Hyla‐Klekot et al. [4]	16 years	Female	Symptoms of SLE occurred concomitantly with anorexia nervosa but the diagnoses of SLE was delayed two years after the diagnosis of anorexia nervosa	Fever Skin rash Arthritis Gastroduodenitis Leukopenia, thrombocytopenia Autoimmune antibodies (ANA, anti dsDNA, anti‐SS‐A, anti‐Ro‐52, anti‐nucleosome, anti‐histone and anti‐ribosomal P antibodies) Lupus nephritis Raynaud's phenomenon	Initial admission to a psychiatric hospital for anorexia nervosa management and nutritional support Excellent response to immunosuppressive therapy (steroids, chloroquine phosphate, and mycophenolate mofetil) in terms of SLE disease activity and anorexia nervosa (mood, behavior, and weight)
Bambery et al. [8]	13 years	Female	One year after the diagnosis of SLE	Fever Vasculitis rash Alopecia pancytopenia Autoimmune antibodies (ANA, anti dsDNA, low C3 and C4)	Initial weight gain due to steroid use followed by strict dieting and exercise leading to weight loss Patient recovered with behavioral and psychological therapy as well as an increased dose of steroids.
Xu et al. [7]	13 years	Female	Diagnosis of anorexia nervosa preceded SLE diagnosis by a short interval	Depression Autoimmune antibodies (ANA, Anti dsDNA) Anemia, neutropenia Elevated liver enzymes	Initial bone marrow examination showed lupus cells followed by SLE investigations and diagnosis Excellent response to methylprednisolone
Toulany et al. [5]	Mean: 13.6 years (Range 11.2 to 16.8 years)	All females	6 patients had onset of anorexia nervosa around 20 months before SLE diagnosis1 patient diagnosed with anorexia nervosa after 15 months of SLE diagnosis	Arthritis (all patients) Autoimmune antibodies: ANA (N = 7), anti dsDNA (N = 2), anti‐Ro (N = 2), anti‐La (N = 1), rheumatoid factor (N = 1), anti‐Sm and anti RNP (N = 1) Malar rash Lymphopenia Raynaud phenomenon CNS involvement Depression, anxiety, paranoia	The single case of anorexia nervosa following SLE diagnosis was attributed to steroids use and cushingoid features Out of the sex patients who had anorexia nervosa diagnosis before SLE, one patient died of invasive cytomegalovirus infection and the remaining five case had resolution of anorexia nervosa symptoms with SLE treatment Two patients had relapse of anorexia nervosa with steroids tapering

In this case, the patient's age was older compared to previously reported cases, which is atypical for the onset of anorexia nervosa. A large cohort study based on a representative sample from the United States reported a median onset age of 17.4 years (IQR: 15.2–20.5 years), with a significant female predilection (AOR = 12) [17]. The uncommon presentation of anorexia nervosa symptoms in a woman of reproductive age, along with systemic inflammatory manifestations (skin rash, alopecia, proteinuria, and pancytopenia), raised clinical suspicion of SLE. Sirufo et al. suggested that anorexia nervosa and autoimmune diseases might interact through proinflammatory cytokines, the brain‐gut‐microbiota axis, autoantibody production, and shared immunological pathways [10]. Additionally, a genome‐wide association study of 3,495 anorexia nervosa cases identified a region on chromosome 12 linked to autoimmune diseases [18]. However, the pathophysiology underlying comorbid anorexia nervosa in SLE may differ depending on whether the anorexia nervosa onset occurs before or after the diagnosis and treatment of SLE [5, 8]. While elevated inflammatory cytokines and immune dysregulation may trigger anorexia nervosa in undiagnosed SLE patients, steroid treatment—with its impact on weight and cushingoid features—could induce anorexia nervosa symptoms in predisposed individuals [10, 11]. Nevertheless, due to the lack of strong evidence regarding the potential association between these conditions, further research is required to explore this hypothesis.

This case involved SLE, anorexia nervosa, and Graves' disease, all of which can contribute to pancytopenia [12, 19, 20, 21], cardiomyopathy [20, 22], and weight loss [1, 10, 23]. Effective management of these overlapping conditions is critical to preventing complications.

To the best of our knowledge, this is the second case of anorexia nervosa presenting as an early symptom in an adult SLE patient. This case underscores several clinical points. First, the coexistence of anorexia nervosa and SLE, and the complete resolution of anorexia nervosa symptoms with immunosuppressive therapy suggest shared autoimmune mechanisms, warranting further research. Second, SLE should be considered in patients with new‐onset neuropsychiatric symptoms and systemic manifestations, particularly in those at epidemiological risk for SLE. Third, early recognition and management of the multiorgan involvement of SLE and its associated autoimmune and inflammatory conditions are vital for complete recovery and optimal outcomes.

## Author Contributions

**Maen Nizam Baroudi:** conceptualization; data curation; writing–original draft; writing–review & editing. **Anwar I Joudeh:** conceptualization; data curation; methodology; writing–original draft; writing–review & editing. **Mohammed Kays Alattiya:** data curation; Investigation; writing–original draft. **Abdo Qaid Ahmed Lutf:** data curation; supervision; writing–review & editing. **Hassan Abuzaid:** conceptualization; methodology; supervision; writing–review & editing. **Salah Mahdi:** conceptualization; project administration; supervision; writing–review & editing.

## Ethics Statement

This work was conducted in accordance with the Declaration of Helsinki (1964). Written informed consent was taken from the patient. This case report was approved by the Institutional Review Board at Hamad Medical Corporation, Doha, Qatar (reference number: MRC‐04‐24‐314).

## Consent

Written informed consent was obtained from the patient for publication of this case report and any accompanying images. A copy of the written consent is available for review by the Editor‐in‐Chief of this journal.

## Conflicts of Interest

The authors declare no conflicts of interest.

## Data Availability

Data can be obtained from the corresponding author upon request.
